# Gender-related differences in patients with carcinoid syndrome: new insights from an Italian multicenter cohort study

**DOI:** 10.1007/s40618-023-02213-1

**Published:** 2023-10-14

**Authors:** R. M. Ruggeri, B. Altieri, P. Razzore, F. Retta, E. Sperti, G. Scotto, M. P. Brizzi, L. Zumstein, A. Pia, A. Lania, E. Lavezzi, G. Nappo, A. Laffi, M. Albertelli, M. Boschetti, I. Hasballa, A. Veresani, N. Prinzi, S. Pusceddu, S. Oldani, F. Nichetti, R. Modica, R. Minotta, A. Liccardi, G. Cannavale, E. M. Grossrubatscher, M. G. Tarsitano, V. Zamponi, M. C. Zatelli, I. Zanata, R. Mazzilli, M. Appetecchia, M. V. Davì, V. Guarnotta, E. Giannetta, A. La Salvia, G. Fanciulli, P. Malandrino, A. M. Isidori, A. Colao, A. Faggiano, NIKE Group

**Affiliations:** 1 Endocrinology Unit, Department of Human Pathology of Adulthood and Childhood DETEV, University of Messina, 98125 Messina, Italy; 2 Division of Endocrinology and Diabetes, Department of Internal Medicine, University of Würzburg, Würzburg, Germany; 3 SC Endocrinologia, Diabetologia e Malattie del Metabolismo, AO Ordine Mauriziano di Torino, Largo Turati, 62 10128 Turin, Italy; 4 SCDU Oncologia, AO Ordine Mauriziano di Torino, Largo Turati, 62 10128 Turin, Italy; 5 Department of Oncology, University Hospital San Luigi Gonzaga, University of Turin, Orbassano, Turin, Italy; 6 Internal Medicine, Department of Clinical and Biological Sciences, S. Luigi Hospital, University of Turin, Turin, Italy; 7 Department of Biomedical Sciences, Humanitas University, 20089 Pieve Emanuele, Italy; 8 Endocrinology, Diabetology and Andrology Unit, IRCCS Humanitas Research Hospital, 20089 Rozzano, Italy; 9 Pancreas Surgery Unit, IRCCS Humanitas Research Hospital, 20089 Rozzano, Italy; 10 Medical Oncology and Hematology Unit, Humanitas Cancer Center, IRCCS Humanitas Research Hospital, Rozzano, Milan Italy; 11 Endocrinology Unit, Department of Internal Medicine and Medical Specialties (DIMI), University of Genova, 16132 Genoa, Italy; 12 Endocrinology Unit, IRCCC Ospedale Policlinico San Martino, 16132 Genoa, Italy; 13 Medical Oncology, Foundation IRCCS National Cancer Institute, Milan, Italy; 14 First Department of Internal Medicine, San Matteo Hospital Foundation, Padua, Italy; 15 Endocrinology, Diabetology and Andrology Unit, Department of Clinical Medicine and Surgery, Federico II University of Naples, Naples, Italy; 16 Endocrine Unit, ASST Grande Ospedale Metropolitano Niguarda, Milan, Italy; 17 Department of Medical and Surgical Sciences, University Magna Graecia of Catanzaro, Catanzaro, Italy; 18 Endocrinology Unit, Department of Clinical and Molecular Medicine, Sant’Andrea Hospital, Sapienza University of Rome, ENETS Center of Excellence, Rome, Italy; 19 Section of Endocrinology, Geriatrics and Internal Medicine, Department of Medical Sciences, University of Ferrara, Ferrara, Italy; 20 Oncological Endocrinology Unit, IRCCS Regina Elena National Cancer Institute, Rome, Italy; 21 Department of Medicine, Section of Endocrinology, University and Hospital Trust of Verona, Verona, Italy; 22 Dipartimento di Promozione della Salute, Materno-Infantile, Medicina Interna e Specialistica di Eccellenza “G. D’Alessandro” (PROMISE), Sezione di Malattie Endocrine, del Ricambio e della Nutrizione, Università di Palermo, 90127 Palermo, Italy; 23 Department of Experimental Medicine, Sapienza University of Rome, Rome, Italy; 24 Division of Medical Oncology 2, IRCCS Regina Elena National Cancer Institute, 00144 Rome, Italy; 25 Neuroendocrine Tumour Unit, Department of Medicine, Surgery and Pharmacy, University of Sassari-Endocrine Unit, AOU Sassari, Sassari, Italy; 26 Endocrinology Unit, Department of Clinical and Experimental Medicine, University of Catania and Garibaldi, Nesima Medical Center, Catania, Italy; 27 UNESCO Chair on Health Education and Sustainable Development, Federico II University, 80138 Naples, Italy

**Keywords:** Carcinoid syndrome, Neuroendocrine tumors, Neuroendocrine neoplasm, Gender, Gender medicine, Prognosis, Sex

## Abstract

**Background:**

The incidence of neuroendocrine neoplasm (NEN) and related carcinoid syndrome (CaS) has increased markedly in recent decades, and women appear to be more at risk than men. As per other tumors, gender may be relevant in influencing the clinical and prognostic characteristics of NEN-associated CS. However, specific data on carcinoid syndrome (CaS) are still lacking.

**Purpose:**

To evaluate gender differences in clinical presentation and outcome of CaS.

**Methods:**

Retrospective analysis of 144 CaS patients from 20 Italian high-volume centers was conducted. Clinical presentation, tumor characteristics, therapies, and outcomes (progression-free survival, PFS, overall survival, OS) were correlated to gender.

**Results:**

Ninety (62.5%) CaS patients were male. There was no gender difference in the site of primary tumor, tumor grade and clinical stage, as well as in treatments. Men were more frequently smokers (37.2%) and alcohol drinkers (17.8%) than women (9.5%, *p* = 0.002, and 3.7%, *p* = 0.004, respectively). Concerning clinical presentation, women showed higher median number of symptoms (*p* = 0.0007), more frequent abdominal pain, tachycardia, and psychiatric disorders than men (53.3% vs 70.4%, *p* = 0.044; 6.7% vs 31.5%, *p* = 0.001; 50.9% *vs*. 26.7%, *p* = 0.003, respectively). Lymph node metastases at diagnosis were more frequent in men than in women (80% vs 64.8%; *p* = 0.04), but no differences in terms of PFS (*p* = 0.51) and OS (*p* = 0.64) were found between gender.

**Conclusions:**

In this Italian cohort, CaS was slightly more frequent in males than females. Gender-related differences emerged in the clinical presentation of CaS, as well as gender-specific risk factors for CaS development. A gender-driven clinical management of these patients should be advisable.

## Introduction

Gender medicine is an emerging field aiming to study differences between men and women in terms of disease prevention and outcome, clinical manifestations and therapy response [1]. The main goal of gender medicine is to understand the mechanisms underlying gender-related differences, to provide a tailored management of the patient [1]. This approach has been studied in many medical fields. More recently, scientific attention has been focused on gender impact on oncological pathologies, including neuroendocrine neoplasms (NEN) [2–4].

The incidence of NEN markedly increased worldwide in recent decades, with slightly higher prevalence in females. NEN-related carcinoid syndrome (CaS) incidence has also increased in the last years, mostly in women [5, 6]. CaS is a complex and heterogeneous disorder caused by increased secretion of several humoral substances, the most prominent being serotonin (5-hydroxytryptamine, 5-HT), but also including histamine, and kinins. The clinical presentation of CaS is very heterogeneous, likely in relation to variable secretion of the different substances at the basis of the syndrome [7, 8], ranging from mild, often misdiagnosed, symptoms such as mild diarrhea and flushing, to serious clinical manifestations deeply impacting prognosis and quality of life, such as uncontrolled diarrhea and fibrosis complications [9]. As a whole, CaS is associated with an overall reduced survival [9].

Literature on gender differences in this field is very limited, and available data are at least partially inconsistent [4, 10–12]. As in other tumors, gender might be relevant in influencing the clinical characteristics presentation and prognosis of NEN-related CaS, providing an intriguing tool to obtain more effective and safe therapeutic strategies, tailored on patient’s characteristics. The aim of the present study was to investigate gender influence on CaS in terms of prevalence, clinical manifestations, prognosis and response to therapy in a large multicenter cohort of Italian patients.

## Materials and methods

This is a large retrospective, observational, multicenter study conducted in patients affected by NEN-associated CaS that was diagnosed and followed up between 2000 and 2022. The study involved 20 Italian referral centers for NENs, in the context of the Neuroendocrine Tumors Innovation in Knowledge and Education (NIKE) project. The inclusion criteria were: (1) cytological or histological diagnosis of NEN, according to the 2022 WHO classification, and (2) CaS syndrome, defined as the presence of chronic diarrhea and/or flushing after excluding other potential causes, and confirmed by increased 24 h urinary 5-hydroxy-indole-acetic acid concentrations. Patients younger than 18 years old were excluded from the study.

Data were collected anonymously from each center using a specific database, subdivided into different sections. The first section of the database referred to clinical data at diagnosis including gender, age, clinical manifestations (presence and frequency of flushing and diarrhea, presence of abdominal pain, tachycardia, etc.), tumor site and disease status (localized vs metastatic disease). Other specific items were the presence of comorbidities, including obesity, type 2 diabetes mellitus (T2DM), hypertension and major cardiovascular events (myocardial infarction, stroke), smoking and drinking status defined according to the Center for Disease Control and Prevention (CDC)—National Center for Health Statistics (NCHS) glossary [13], as previously reported [3]. The second section regarded pathological features according to the 2022 NENs WHO classification [14]. Proliferative activity by immunostaining for Ki67 antigen and grading in tumor tissue were considered. Tumor staging was defined according to AJCC/TNM 8th ed. [15]. A third session covered the type of treatment, including surgical resection of the primary tumor, loco-regional therapies of metastases (transarterial chemoembolization, selective internal radiotherapy and surgical resection of metastases), medical therapies (including somatostatin analogues, everolimus, sunitinib, chemotherapy, telotristat) and peptide receptor radionuclide therapy (PRRT). Follow-up data and clinical outcome in terms of progression-free survival (PFS) and overall survival (OS) from the diagnosis of CaS were evaluated.

The study was conducted in accordance with the Declaration of Helsinki. Approval was obtained from the local ethics research committees (protocol number 03/22), and the subjects were enrolled after providing their informed consent for using anonymized data.

### Statistical analysis

Distribution of continuous variables was evaluated by the Shapiro–Wilk test. Parametric t-test or non-parametric Mann–Whitney test were used for the comparison of continuous variables, whereas the Fisher’s exact test or the Chi-square (χ^2^) was used for categorical variables, as appropriate. Progression-free survival (PFS) was calculated from the time of diagnosis of NEN to the first radiological evidence of tumor relapse, or to the last follow-up in patients without tumor progression. Overall survival (OS) was calculated from the diagnosis of NEN to patient death or last follow-up or the end of data collection. Kaplan–Meier method with log-rank test was used to analyze cumulative survival considering the entire cohort of patients. Univariate and multivariate analyses were performed by Cox proportional hazards regression model, evaluating hazard ratio (HR) and 95% confidence interval (CI). In the multivariate model, we included all the parameters with a *p* value less than 0.10 on univariate regression analysis. Statistical analysis was performed using SPSS Software (Version 29.0, SPSS Inc., Chicago, IL, USA) and GraphPad Prism (version 9.0, La Jolla, CA, USA) and a *p* value < 0.05 was considered statistically significant.

## Results

### Characteristics of patients

A total of 144 patients with diagnosis of NEN and CaS were included in the study. Among these, 90 (62.5%) were men and 54 (37.5%) were women. All patients had a sporadic NEN. General and gender-related clinical and pathological characteristics of the entire cohort are shown in Table 1. Age at diagnosis was not different between genders (median age 59 years, range 23–82, in women, and 60 years, range 19–84, in men, *p* = 0.33). No significant difference was found considering the onset of CaS, although CaS occurred after NEN diagnosis most frequently in men (40%) than in women (25%).

**Table 1:** Clinical and pathological characteristics of the entire cohort and according to gender

Parameters	All patients	Women	Men	*p*	Chi- square
N of patients	144	54	90	–	–
Age at diagnosis of NEN, years	60 (19–84)	59 (23–82)	60 (19–84)	0.33	–
Age at diagnosis of CaS, years	61 (23–84)	60 (23–84)	61 (26–83)	0.28	–
Time between symptoms onset and CaS diagnosis, months	6 (0–360)	5 (0–120)	6 (0–360)	0.75	–
*Onset of CaS*:				0.20	3.19
At NEN diagnosis	59 (41.0%)	24 (44.4%)	35 (38.9%)		
After NEN diagnosis	50 (34.7%)	14 (25.9%)	36 (40.0%)		
Before NEN diagnosis	35 (24.3%)	16 (29.6%)	19(21.1%)		
*Primary tumor site*:				0.74	1.26
Pancreas	9 (6.3%)	3 (5.6%)	6 (66.7%)		
Intestinal tract	114 (79.2%)	42 (77.8%)	72 (80.0%)		
Lung	14 (9.7%)	7 (13.0%)	7 (7.8%)		
Unknown primary	7 (4.9%)	2 (3.7%)	5 (5.6%)		
Tumor grade of the primary tumor or synchronous metastasis*:				0.18	4.91
G1	77 (53.5%)	23 (42.6%)	54 (60.0%)		
G2	56 (38.9%)	26 (48.1%)	30 (33.3%)		
G3	3 (2.1%)	2 (3.7%)	1 (1.1%)		
Unknown	8 (5.6%)	3 (5.6%)	5 (5.6%)		
Ki67%	2 (0–28)	3 (1–28)	2 (0–20)	0.14	–
*Metastases at NEN diagnosis*:				0.18	1.11
Yes	129 (89.6%)	46 (85.2%)	83 (92.2%)		
No	15 (10.4%)	8 (14.8%)	7 (7.8%)		
*Disease status at diagnosis of CaS*:				0.29	1.11
Localized disease	3 (2.1%)	2 (3.7%)	1 (1.1%)		
Metastatic disease	141 (97.9%)	52 (36.9%)	89 (98.9%)		
*Liver metastasis*:				0.36	0.85
Yes	120 (83.3%)	43 (79.6%)	77 (85.6%)		
No	24 (16.7%)	11 (20.4%)	13 (14.4%)		
*Lung metastasis*:				0.44	0.60
Yes	15 (10.4%)	7 (13.0%)	8 (8.9%)		
No	129 (89.6%)	47 (87.0%)	82 (91.1%)		
*Lymph node metastasis*:				0.04	4.08
Yes	107 (74.3%)	35 (64.8%)	72 (80.0%)		
No	37 (25.7%)	19 (35.2%)	18 (20.0%)		
*Bone metastasis*:				0.60	0.28
Yes	30 (20.8%)	10 (18.5%)	20 (22.2%)		
No	114 (79.2%)	44 (81.5%)	70 (77.8%)		
*Other metastasis*:				0.53	0.38
Yes	41 (28.5%)	17 (31.5%)	24 (26.7%)		
No	103 (71.5%)	37 (68.5%)	66 (73.3%)		
N of site of distant metastasis	2 (0–5)	2 (0–5)	2 (0–5)	0.44	–

Continuous variables were reported as median (minimum – maximum range) and categorical variables as numbers (percentages)

*CaS* carcinoid syndrome, *G* grading, *N* number, *NEN* neuroendocrine neoplasm, *if the grading of the primary tumor was not available

**Fig. 1:**
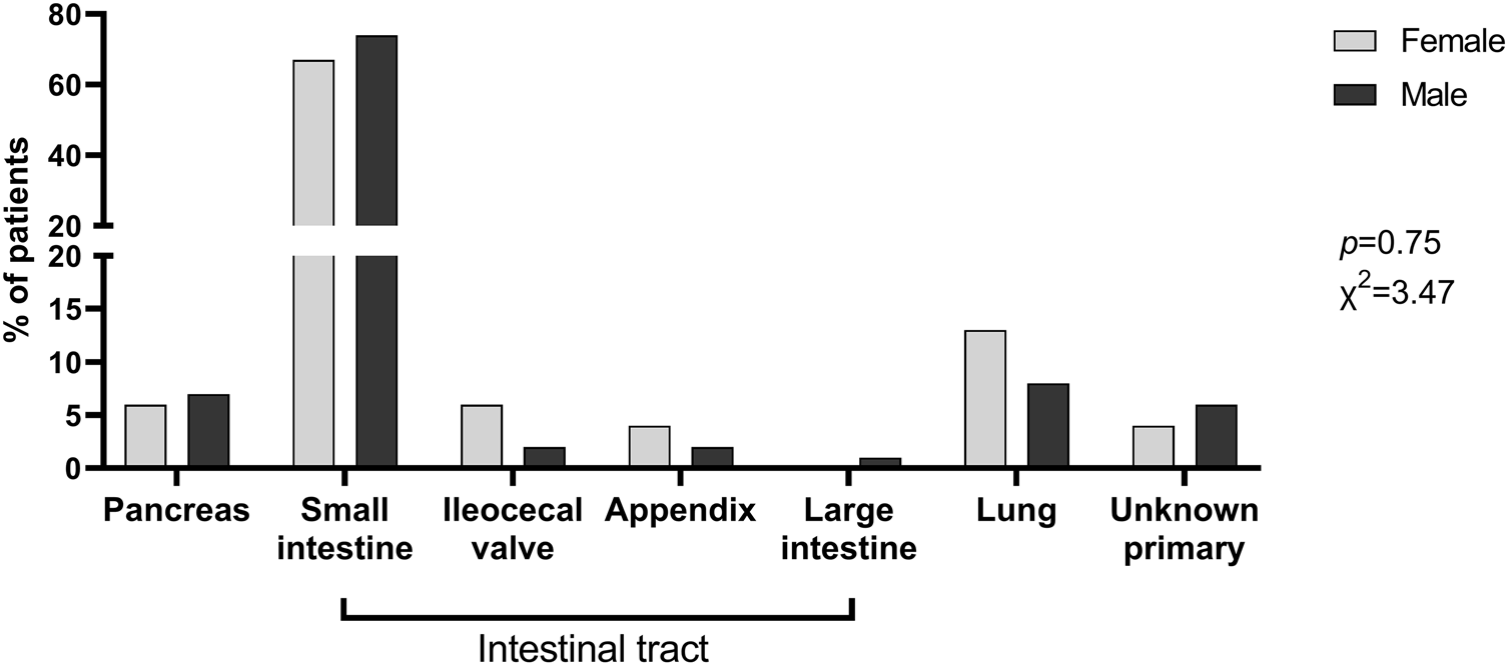
Primary tumor site according to gender. No significant difference was observed in terms of site of the primary tumor between female and male patients, with neoplasia of the intestinal tract being the most frequent in both sexes. Histograms represent the percentage of patients. Statistical analysis by Chi-square test on absolute values

No differences were observed in terms of site of primary tumor (χ^2^ = 3.47,* p* = 0.75) (Fig. 1), with NEN of the intestinal tract being more prevalent in both genders (80% and 77.8% in men and women, respectively). Moreover, tumor grade and disease status did not differ between genders, with almost all cases (n = 141, 97.9%) metastatic disease at the time of CaS diagnosis (Table 1). Liver was the most frequent site of metastases in both genders (79.6% in women and 85.6% in men), while loco-regional lymph node metastases were more frequently observed in male than female patients (80% vs. 64.8%, HR = 1.38, 95%CI 097–1.97, *p* = 0.04) at diagnosis. Other distant metastases, including metastases of peritoneum and pleura, as well as bone metastases were also frequently reported in our cohort, whereas lung metastases were observed in 10.4% of the entire population, without differences between women and men (Table 1).

### Clinical presentation of CaS

**Fig. 2:**
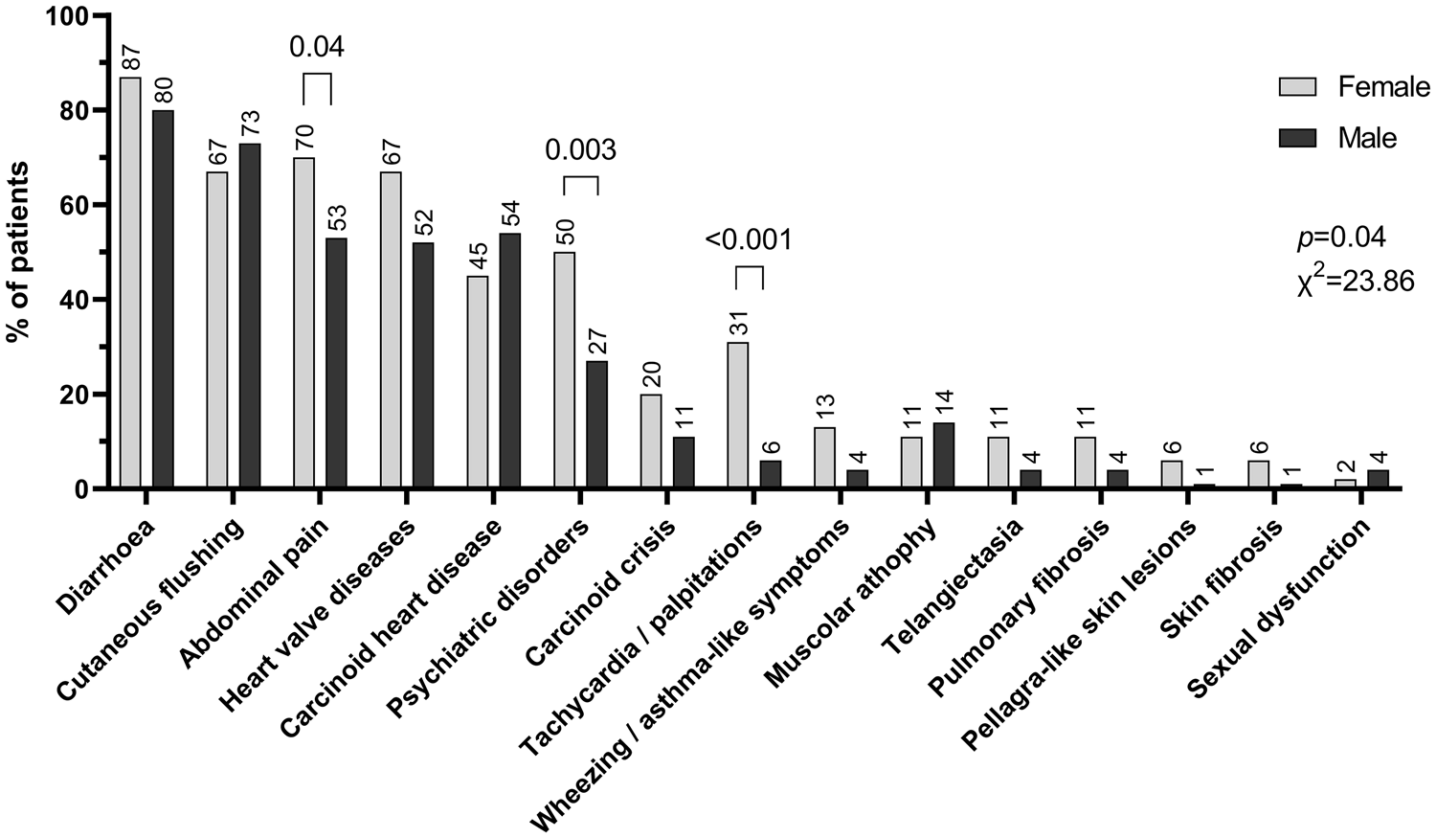
Clinical presentation of carcinoid syndrome according to gender. Clinical presentation of CaS differed between genders, with women reporting a significantly higher frequency of abdominal pain, psychiatric disorders, and tachycardia. No significant differences were observed among the other symptoms, including diarrhea and cutaneous flushing, which were the most frequently reported symptoms on both sexes. Histograms represent the percentage of patients. Statistical analysis by Chi-square test on absolute values

Clinical presentation of CaS was different between genders (Fig. 2). Women showed a significantly higher median number of symptoms than men (5, range 1–13, *vs*. 3, range 1–13, *p* = 0.0007). The most frequent presentation of CaS was diarrhea in both genders (*n* = 47, 87.0%, of female and *n* = 72, 80%, of male patients), with 65.0% of women reporting a slightly higher frequency of at least four bowel motions per day than men (49.2%, *p* = 0.11; Fig. 2). Abdominal pain was the second symptom in women (*n* = 38, 70.4% of cases) and was significantly more frequent than in male (*n* = 48, 53.5%, HR = 1.60, 95%CI 0.99–2.59, *p* = 0.04), whereas cutaneous flushing was the second most frequent symptom in men (*n* = 66, 73.3%, *vs*. *n* = 36, 36.7%, in women) (Fig. 2). Frequency of flushing episodes did not differ between genders. Almost half of the patients (46.6% female and 47.7% male) reported rare flushing episodes, while 31.5% of women and 31.8% of men referred to more than two episodes per day (*p* = 0.98). Compared to male patients, female patients reported a significantly higher frequency of palpitations (31.5% *vs*. 6.7%, HR = 2.42, 95%CI 1.68–3.47, *p* < 0.001) and psychiatric disorders, including depression (50.9% *vs*. 26.7%, HR = 1.87, 95%CI 1.24–2.84, *p* = 0.003; Fig. 2). Moreover, women showed a higher, but not significant increased risk of wheezing and asthma-like symptoms than men (13.0% *vs*. 4.4%, HR = 1.80, 95%CI 1.09–2.98, *p* = 0.06). A similar trend was observed also for carcinoid crises, which were slightly more frequent in female than male patients (20.4% vs. 11.4%, HR = 1.47, 95%CI 0.92–2.37, *p* = 0.14). No differences between genders were found in the other symptoms, which were reported in less than 15% of the entire cohort (Fig. 2). Since most of our female patients were in menopausal age, a role of estrogen exposure or deprivation cannot be clearly established for such a difference in clinical presentation.

Data of heart valve diseases and carcinoid heart disease were available in a total of 130 and 133 cases, respectively, and were found in a similarly large proportion of both female and male patients (67.3% vs. 51.9% of cases for heart valve diseases, respectively, and 44.9% vs*.* 54.2% cases for carcinoid heart disease, respectively; *p* > 0.05) (Fig. 2). In patients with heart valve diseases, no gender differences were observed in the number of affected valves (median 1, range 1–3, in women, and median 1, range 1–4, in men, *p* = 0.31), as well as in the affected site, with the tricuspid valve being the most commonly affected valve in both genders (75.8% of women and 83.3% of men, *p* = 0.42), followed by the mitral valve (39.4% of women and 38.1% of men, *p* = 0.91). Disease of the aortic and pulmonary valves was observed in 21.2% and 15.2%, respectively, of women, and 23.8% (*p* = 0.79) and 26.2% (*p* = 0.25), respectively, of men. No difference was observed in terms of severity of heart valve disease between genders, with most female (46.9%) and male patients (47.6%) having moderate dysfunction (*p* = 0.43). Also considering the cases with carcinoid heart disease, no difference was found in terms of left ventricle ejection fraction between female and male patients (median 60%, range 40–79, vs*.* 58%, range 28–75, respectively, *p* = 0.19).

### Comorbidities and risk factors

Data on BMI were available in 135 patients (51 females and 84 males) (Table 2). Most patients had normal body weight (BMI 18–25 kg/m^2^) without significant differences between female and male patients (51.9% of female and 48.9% of male, χ^2^ = 4.70, *p* = 0.32), even though median BMI was significantly higher in men (24.1 kg/m^2^, range 17.0–36.3) than women (22.9 kg/m^2^, range 15.0–32.0, *p* = 0.02).

**Table 2:** Comorbidities and risk factors in the entire cohort and according to gender

Parameters	All patients (*n* = 144)	Women (*n* = 54)	Men (*n* = 90)	*p*	Chi- square
BMI, kg/m^2^	24 (15.0–36.3)	22.9 (15.0–32.0)	24.1 (17.0–36.3)	0.02	–
*BMI categories*:				0.32	4.70
< 18 kg/m^2^	7 (4.9%)	5 (9.3%)	2 (2.2%)		
≥ 18–25 kg/m^2^	72 (50.0%)	28 (51.9%)	44 (48.9%)		
≥ 25–30 kg/m^2^	47 (32.6%)	16 (29.6%)	31 (34.4%)		
≥ 30 kg/m^2^	9 (6.3%)	2 (3.7%)	7 (7.8%)		
Unknown	9 (6.3%)	3 (5.6%)	6 (6.7%)		
*T2DM*:				0.09	2.82
Yes	26 (18.1%)	6 (11.1%)	20 (22.2%)		
No	118 (81.9%)	48 (88.9%)	70 (77.8%)		
*Major cardiovascular event*:				0.008	7.15
Yes	11 (7.6%)	0 (0%)	11 (12.2%)		
No	133 (92.4%)	54 (100%)	79 (87.8%)		
*Hypertension*:				0.07	5.05
Yes	71 (49.3%)	21 (38.9%)	50 (55.6%)		
No	72 (50.0%)	32 (59.3%)	40 (44.4%)		
Unknown	1 (0.7%)	1 (1.9%)	0		
Smoking status*:				0.003	13.71
Never smoker	102 (70.8%)	48 (88.9%)	54 (60.0%)		
Former smoker	22 (15.3%)	3 (5.6%)	19 (21.1%)		
Current smoker	15 (10.4%)	2 (3.7%)	13 (14.4%)		
Unknown	5 (3.5%)	1 (1.9%)	4 (4.4%)		
Drinking status**:				0.04	6.23
Abstainer/infrequent	126 (87.5%)	52 (96.3%)	74 (82.2%		
Drinker	16 (11.1%)	2 (3.7%)	14 (15.6%)		
Former drinker	2 (1.4%)	0 (0%)	2 (2.2%)		

Continuous variables were reported as median (minimum – maximum range) and categorical variables as numbers (percentages)

*BMI* body mass index, *T2DM* type 2 diabetes mellitus

*Smoking status was categorized as “current smoker”, when patient currently smokes cigarettes, “former smoker”, when patient has smoked at least 100 cigarettes in his or her lifetime but who had quit smoking at the time of interview, and “never smoked ^3,16^. **Drinking status was defined as follows: “current drinker”, “lifetime abstainer” and “former drinker”, when patient had drinks in the past year ^3,16^

Women did not report any major cardiovascular events, which were observed in 12.2% of men (HR = 1.68, 95%CI 1.46–1-94, *p* = 0.008; Table 2). Moreover, men showed a trend to an increased frequency of T2DM and hypertension than women (22.2% *vs*. 11.1%, HR = 1.30, 95%CI 1.00–1.68, *p* = 0.09 and 55.6% *vs*. 39.6%, HR = 1.27, 95%CI 0.98–1.64, *p* = 0.07, respectively; Table 2). Men were more frequently former or current smokers (35.5%) and current or former alcohol drinkers (17.8%) than women (9.3%, *p* = 0.003, and 3.7%, *p* = 0.04, respectively; Table 2).

### Therapies

**Fig. 3:**
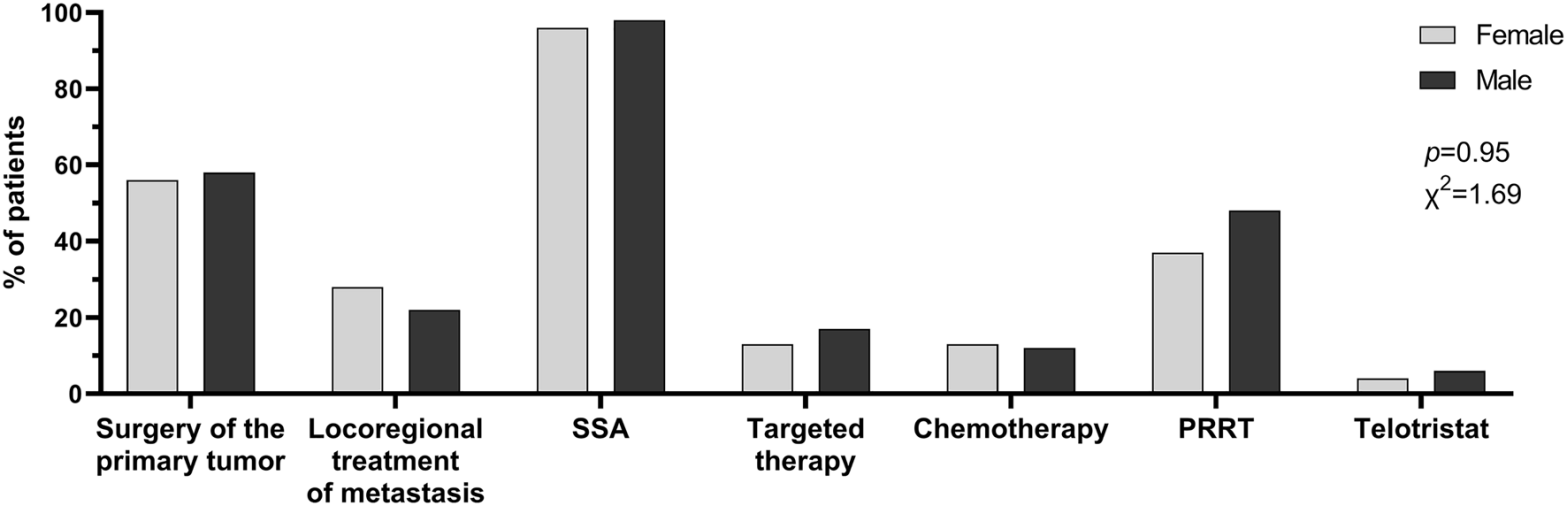
Treatment according to gender. The type of treatment was not different between female and male patients, with the somatostatin analogs (SSA) being the most used therapy in both genders, followed by surgery resection of the primary tumor and by peptide receptor radionuclide therapy (PRRT). Histograms represent the percentage of patients. Statistical analysis by Chi-square test on absolute values

No differences were observed between women and men in terms of the type of treatment (χ^2^ = 1.69, *p* = 0.95; Fig. 3). Surgery of the primary tumor was performed in 55.6% (*n* = 30) of women and 57.8% (*n* = 52) of men (χ^2^ = 0.07, *p* = 0.79). Loco-regional treatment of metastases was performed in 27.8% (*n* = 15) of female and 22.2% (*n* = 20) of male patients (χ^2^ = 0.57, *p* = 0.45).

Except for four cases, most patients (96.3% of women and 97.8% of men) were treated with SSA (χ^2^ = 0.27, *p* = 0.60). Particularly, 22 (42.3%) females and 45 (51.1%) males had the initial SSA treatment modified during the follow-up, switching to a high-dose SSA schedule, or shortening the time between SSA injections, or switching the type of the analog. SSA therapy was efficacious for the control of CaS symptoms in 73.1% of women and 80.9% of men since the beginning of the treatment and in the other 9.9% and 5.6% female and male patients, respectively, after SSA modification. Considering the entire duration of the SSA therapy, the median time of SSA efficacy for the control of the CaS symptoms was similar in both genders (18.5 months in women and 17 months in men; *p* = 0.83). Other medical therapies including targeted therapies (everolimus or sunitinib) and different chemotherapies (temozolomide, or capecitabine, or capecitabine plus temozolomide, or oxaliplatin plus capecitabine, or 5-fluorouracil, or carboplatin) were administered in 15.3% and 12.5% of cases, respectively, without differences between male and female patients (Fig. 3). Twenty-five pe cent of women and 23.6% of men were treated with two or more lines of medical therapy (including SSA, targeted therapy and chemotherapy). PRRT was used slightly more often in men than women (47.8% vs. 37%, respectively), although the difference was not statistically significant (χ^2^ = 01.58, *p* = 0.21; Fig. 3). Finally, telotristat was administrated in only 3.7% (*n* = 2) of women and 5.6% (*n* = 5) of men (χ^2^ = 0.25, *p* = 0.62).

### Survival

**Fig. 4:**
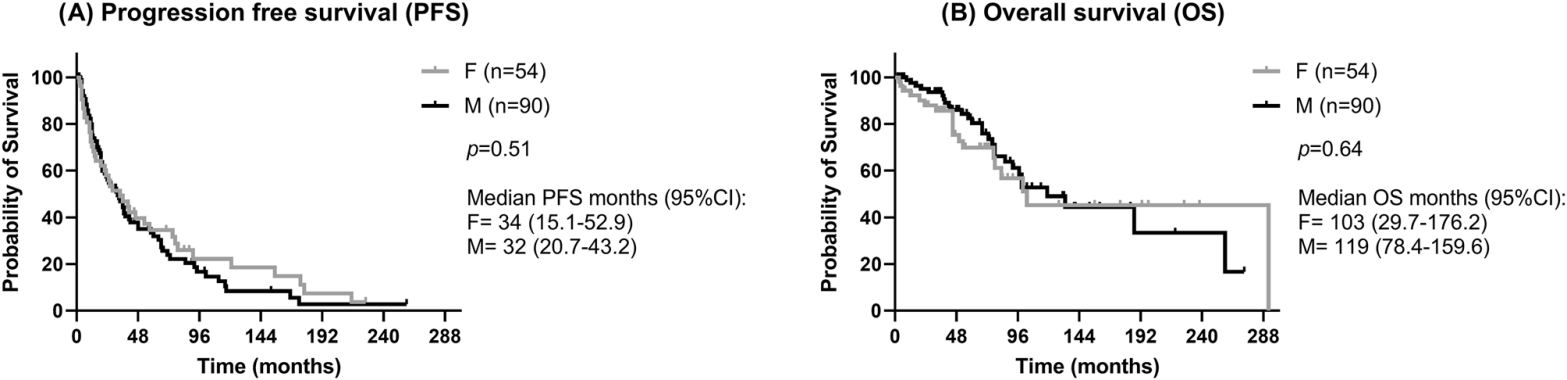
Clinical outcome according to gender. Progression-free survival (**A**) and overall survival (**B**) did not differ between female and male patients. Statistical analysis by Kaplan–Meier survival curves and log rank test

PFS did not differ between genders (Fig. 4A), with women having a median PFS of 34 months (95%CI 15.1–52.9) and men a median PFS of 32 months (95%CI 20.7–43.2).). One-, 2- and 5-year PFS rates were 70.3%, 55.8%, and 35.3%, respectively, in women and 73.8%, 56.0%, and 34.6%, respectively, in men. By stratifying the patients according to gender, only the use of two or more medical treatments was slightly associated with worse PFS particularly in women (*p* = 0.62), where the median PFS in patients with two or more medical treatment compared to those treated with only one medical therapy was 22 vs. 34 months.

Considering the entire cohort, only a higher number of medical treatments (≥ 2) slightly correlate with a worse PFS (HR = 1.46, 95%CI 0.61–1.46) at univariate analysis (Table 3**)**. Multivariate analysis could not be performed.

**Table 3:** Cox regression univariate and multivariate analysis of prognostic factors influencing progression-free survival

Parameter	*n*	Univariate PFS		Multivariate PFS	
		*p*	HR (95%CI)	*p*	HR (95%CI)
*Sex*:		Ref			
F	53	0.34	1.22 (0.81–1.86)	–	
M	87				
*Age at CaS diagnosis*:		Ref			
< 61 years	69	0.12	1.38 (0.92–2.07)	–	
≥ 61 years	71				
*Primary tumor site*:					
Pancreas	9	Ref		–	
Intestinal tract	111	0.41	1.42 (0.62–3.27)		
Lung	13	0.67	1.28 (0.41–3.99)		
Unknown primary	7	0.80	0.85 (0.24–3.03)		
*Grading*:					
G1	75	Ref		–	
G2–G3	57	0.45	1.17 (0.77–1.78)		
*Onset of CaS*:					
At NEN diagnosis	58	Ref		Ref	
After NEN diagnosis	48	0.02	1.69 (1.07–2.67)	0.07	1.57 (0.96–2.43)
Before NEN diagnosis	34	0.60	0.87 (0.51–1.48)	0.82	0.94 (0.55–1.61)
*Liver metastasis*:				–	–
No	24	Ref	Ref		
Yes	116	0.59	1.17 (0.66–2.06)		
*Lung metastasis*:					
No	125	Ref	Ref	–	
Yes	15	0.53	1.25 (0.62–2.49)		
*Lymph node metastasis*:					
No	36	Ref	Ref	–	
Yes	104	0.82	0.94 (0.58–1.52)		
*Bone metastasis*:					
No	112	Ref	Ref	Ref	
Yes	28	0.051	1.61 (0.10–2.61)	0.27	1.32 (0.80–2.16)
*Number of medical therapies*:					
0–1 line	103	Ref	Ref	–	
≥ 2 medical therapies	34	0.18	1.34 (0.87–2.07)		
*PRRT*:					
No	80	Ref	Ref	Ref	
Yes	60	< 0.001	2.07 (1.38–3.11)	0.002	1.90 (1.26–2.88)

Data for progression-free survival (PFS) were available in 140 patients. In the multivariable model were included variables with a *p* value less than 0.10 in the univariate analysis

*CaS* Carcinoid Syndrome, *F* female, *M* male, *n* number of patients, *NEN* neuroendocrine neoplasm, *-* not included in the multivariate analysis

Similar to PFS, also OS did not differ between genders (Fig. 4B) and median OS was similar between women (103 months, 95%CI 29.7–176.2) and men (119 months, 95%CI 78.4–159.6, *p* = 0.64). One-, 2- and 5-year OS rates were 90.1%, 75.3%, and 69.8%, respectively, in women and 97.6%, 93.7%, and 73.5%, respectively, in men. By stratifying the patients according to gender, younger age at NEN diagnosis (< 60 years old) slightly correlated with longer median OS than older age in both female and male patients (*p* = 0.07). Particularly, in younger women the median OS was 292 months (compared to 83 months in older women, whereas younger men had median OS of 258 months compared to 99 months in younger men). Also, tumor grading G2–G3 significantly correlated with a shorter OS (*p* = 0.013), but this was more evident in men than women, with male patients with G2–G3 tumor having a shorter median OS (76 months) than those with G1 tumor (median not reached). A trend to a shorter OS was also observed for the presence of lung metastases in both genders (*p* = 0.09; median OS 53 months vs. 83 months in women with and without lung metastases, respectively, and 92 months vs. 187 months in men with and without lung metastases, respectively).

Considering the entire cohort, G2–G3 tumors (HR = 2.17, 95%CI 0.19–3.95, *p* = 0.01) and presence of lung metastases (HR = 1.32, 95%CI 0.90–4.58, *p* = 0.09) were, respectively, significantly or slightly associated with an increased risk of short OS at univariate analysis (Table 4). At the multivariate analysis, only tumor grading was an independent prognostic factor of OS (HR = 2.36, 95%CI 1.28–4.33, *p* = 0.006), whereas only a trend was observed for the lung metastasis (*p* = 0.50) (Table 4).

**Table 4:** Cox regression univariate and multivariate analysis of prognostic factors influencing overall survival

Parameter	*n*	Univariate PFS		Multivariate PFS	
		*p*	HR (95%CI)	*p*	HR (95%CI)
*Sex*:			1.03 (0.56–1.88)	–	
F	52	Ref			
M	86	0.93			
*Age at CaS diagnosis*:					
< 61 years		Ref		Ref	
≥ 61 years		0.001	2.97 (1.55–5.69)	0.003	2.81 (1.42–5.55)
*Primary tumor site*:		Ref		–	
Pancreas	9	0.59	1.49 (0.35–6.18)		
Intestinal tract	108	0.63	1.56 (0.26–9.51)		
Lung	14	0.55	1.74 (0.29–10.47)		
Unknown primary	7				
*Grading*:					
G1	72	Ref		Ref	
G2–G3	58	0.02	2.04 (0.10–3.80)	0.001	3.07 (1.54–6.09)
*Onset of CaS*:					
At NEN diagnosis	56	Ref		–	
After NEN diagnosis	50	0.27	1.44 (0.75–2.77)		
Before NEN diagnosis	32	0.46	0.73 (0.32–1.67)		
*Liver metastasis*:					
No		23	Ref	Ref	–
Yes		115	0.71	1.22 (0.43–3.43)	
*Lung metastasis*:					
No	124	Ref	Ref	Ref	
Yes	14	0.01	2.83 (1.26–6.38)	0.07	2.44 (0.93–6.45)
*Lymph node metastasis*:					
No	34	Ref	Ref	–	
Yes	104	0.12	0.60 (0.32–1-14)		
*Bone metastasis*:					
No	109	Ref	Ref	–	
Yes	29	0.23	1.53 (0.77–3.05)		
*Number of medical therapies*:					
0–1 line	103	Ref	Ref	–	
≥ 2 medical therapies	32	0.49	1.24 (0.66–2.33)		
*PRRT*:					
No	80	Ref	Ref	Ref	
Yes	58	0.09	0.60 (0.33–1.10)	0.20	0.66 (0.35–1.24)

Data for overall survival (OS) were available in 138 patients. In the multivariable model were included variables with a *p* value less than 0.10 in the univariate analysis

*CaS* carcinoid syndrome, *F* female, *M* male, *n* number of patients, *NEN* neuroendocrine neoplasm, *-* not included in the multivariate analysis

## Discussion

As the incidence of NEN has been increasing worldwide over the last decades, gender differences have emerged in the epidemiology of these neoplasms. Despite variability among studies, NENs prevalence and incidence have increased mostly in women, and female patients with NENs seem to be more likely diagnosed with CaS [10]. In this light, female gender might represent a risk factor for the occurrence of NEN and related CaS. On the other hand, population-based studies have reported a better overall survival in females compared to males among patients with GEP-NENs [16–21]. In support of these clinical findings, there is an increasing body of evidence from pre-clinical studies providing possible explanations for gender differences in NEN tumorigenesis. In particular, the expression of estrogen and progesterone receptors, and related sex hormone signaling pathways, may play a role in such a difference between genders in NEN [22–28]. Overall, a protective effect of estrogens emerges as opposite to a stimulatory effect of androgens in NENs, as well as in other non-reproductive cancers, by means of genetic, epigenetic and hormonal mechanisms [29, 30]. This adds to the influence of the societal and/or behavioral effect of gender roles (diet, smoking, physical activity, alcohol intake, occupational risk factors) [31]. Nevertheless, the influence of gender on the clinical outcomes and diagnostic and therapeutic strategies in NENs is deeply underestimated in clinical practice, and, apart from registry studies, there are very few clinical trials specifically designed to answer this question in the literature. Moreover, none of them was specifically focused on CaS associated with NENs. This multicenter retrospective study was aimed at investigating gender-specific differences related to clinical features, treatments, and outcomes in patients diagnosed with NEN-associated CaS in a real-life scenario.

In our cohort of Italian patients, we found that the prevalence of NEN-related CaS is slightly higher in men than in women, most cases being diagnosed in the 60 s, without significant gender differences in age at diagnosis. Our data are in agreement with the above-mentioned protective effects of estrogens against NEN tumorigenesis [32], even though most of our female patients (90.7%) were in menopause at the time of diagnosis, so that a comparison between pre- and postmenopausal women cannot be done. However, a protective role for estrogen exposure can be proposed, also taking into account the less frequent occurrence of lymph node metastases in female compared to male patients at diagnosis. Conversely, studies from other countries reported an increased prevalence of CaS and a younger age at diagnosis in females compared to males [5, 33], highlighting the large differences in epidemiological analyses on NEN patients. This raises concerns about potential differences related to the geographical region and suggests that environmental factors, along with different genetic backgrounds, may play a certain role in this context, besides the sex-specific hormonal pattern.

Remarkable differences in clinical presentation of Cas emerged from our analysis, despite that the site of origin of the primary tumor was similar between men and women. In our cohort, as expected, the most frequent presentation of CaS was diarrhea with similar frequency in both genders, even though female patients showed a higher (despite not significant) frequency of bowel motions per day. Noteworthy, our female patients experienced a significantly higher frequency of abdominal pain than male patients. Also, females complained more frequently of palpitations and psychiatric disorders. These gender differences should be taken into account in the differential diagnosis of CaS, to avoid missed or delayed NEN diagnosis, since most of these signs and symptoms are more frequent in females than in males in the general population, so that their relationship with CaS could be overlooked [10]. Despite that confounding factors cannot be excluded, the different pattern of clinical presentation of CaS between genders that emerged from our analysis highlights the importance of a gender-driven diagnostic approach to CaS in daily clinical practice. Such a difference in clinical presentation cannot be attributed only to hormonal factors, since most of our female patients were in postmenopausal age. Certainly, besides sex hormone differences, also genetic and molecular disparities between males and females have a role in the differing clinical symptoms and signs. Also, differences in lifestyle between genders as well as the different psychological impact should be taken into account [34].

Indeed, when analyzing differences by gender in risk factors and comorbidities, we found higher rates of cardiovascular disease and a trend toward increase in frequency of T2DM in men than women, without any relationship with tumor grade and/or behavior. This observation is in line with previous evidence, which showed gender differences in cardiovascular disease in pancreatic NEN [2, 3, 35, 36], and in agreement with the postulated association between diabetes and NEN [37–41]. In addition, in our cohort men were more frequently former or current smokers and current or former alcohol drinkers than women. The observation of a higher frequency of smokers and drinkers among men is interesting in the context of the previously reported association of pancreatic NENs with tobacco smoke and alcohol consumption [2, 3, 37], suggesting that gender-specific risk factors (cardiovascular and metabolic disorders, smoking and alcohol habits) for the development of NEN and associated CaS could be identified.

Concerning tumor behavior, the presence of lymph node metastases at CaS diagnosis was reported more frequently in men than in women. Our analysis did not reveal any significant differences in pathological grade, tumor stage, and distant metastases at diagnosis of CaS between men and women. Scarce data exist in the literature providing evidence that female patients are diagnosed with G1 and low G2 NEN more frequently that male patients [2, 3, 42], but data specifically focused on CaS are not available. In our study, the majority of patients were diagnosed with G1–G2 grading, and no statistically significant difference was found related to gender. Accordingly, PFS and OS did not differ between male and female patients. No gender-based differences in therapeutic approaches was found. Evaluating studies of varying intent (not aimed at evaluating gender differences or focused on CaS), women seemed to have improved outcomes, a slight advantage in response to therapy, especially for liver metastases, and better survival compared to men [3, 18–21, 43–46]. However, neither our study nor previous analyses revealed any gender-related differences in treatment modalities, response to therapy, and patient outcomes in NEN patients [4, 42]. However, when evaluating the therapeutic outcomes, limitations of our study should be taken in account, mostly due to its retrospective nature, the relatively small sample size when subdividing patients according to therapy, and the partial overlap of patients between the treatment groups which could not allow a clear separation of therapeutic effects.

Noteworthy, we found significant gender-related differences in clinical presentation of the syndrome and in some oncological outcomes, so that we could speculate that women presented with more frequent and more relevant symptoms and signs of CaS that may result in a more compromised quality of life compared to men, while men experienced a slightly worse disease in terms of the presence of lymph node metastases at diagnosis, for instance, despite no differences in the overall survival.

Despite some limitations mainly due to the retrospective design and the relatively limited number of patients included, especially women of childbearing age, in comparison to the high numbers of registry studies, the present is the first study that focuses on gender differences in NEN-related CaS, analyzing risk factors and comorbidities, clinical presentation, tumor characteristics, and outcomes as a function of patients’ gender in a real-life scenario. The major strengths of our study are: (i) the special focus on CaS that represents the most common functional syndrome related to NENs, but has not been specifically evaluated before in relation to gender; (ii) the real-life scenario of the study that assesses tumor presentation and outcomes in regular clinical practice, thereby reflecting real adherence to treatment/intervention and outcomes; (iii) the multicentric study design, involving several Italian referral centers for NEN management, providing comprehensive and reliable data for the clinical picture of the CaS population in our country. Our data demonstrated a slightly high proportion of males among CaS patients and depicted patterns of clinical manifestations different by gender, as well as specific gender-related risk factors for CaS development. On the other hand, male patients tend to experience a slightly heavier burden of disease, with lymph node metastases at diagnosis being significantly more frequent in male than female patients. Other parameters, including patients´ age, site of tumor origin and rates/site of metastases, tumor stage, applied treatments and response to therapy, outcomes and survival, did not differ between genders.

In the era of precision medicine, these results contribute to get a more complete picture on NEN-related CaS and further highlight the need of studies which, taking into account gender diversity, may ultimately lead to a gender-driven clinical management of these patients.
